# Editorial in This Issue

**DOI:** 10.1590/S1677-5538.IBJU.2016.02.01

**Published:** 2016

**Affiliations:** Divisão de Urologia do A.C. Camargo Cancer Center, Fundação A. Prudente, São Paulo, Brasil

On the *Editor's Comment* section, Prof. Leite, based on more recent knowledge in low grade (Gleason 6: [3+3])prostate cancer, active surveillance protocols, and in recent International Society of Uropathology (ISUP) recommendations, bring to us her more recent impression favoring the omission of the presence of high grade prostatic intraepithelial neoplasia, in the anatomopathological reports of prostatic biopsies. For the patients, the absence of the term “high grade…. neoplasia” for sure, results in less anxiety.

The approach of asymptomatic <1.0 cm lower pole kidney stones is a crucial dilemma in daily practice, always resulting in debate. In the *Difference of Opinion*, Prof. Mazzuchhi, from University of Sao Paulo, is favorable to treat it, (mainly in selected groups of patients). On the other hand, Drs. Ludwig and Matlaga, from the John Hopkins University, advocates to not treat these patients, because three quarters of the asymptomatic ones will not present symptoms and will not require therapeutic interventions, being better conducted under surveillance and delayed intervention, if necessary.

The first Latin American Consensus of Overactive Bladder (OAB) is a supranational collaborative group, that produced two literature reviews about this complexity neuro-urological entity, described in the last decades: Part I: focusing on the definition of OAB, epidemiological aspects, and research aspects of OAB (page 188), and the Part II (page 199), focusing on the therapeutics modalities (for primary treatment and for refractory cases. In page 312, some Brazilian collaborators of the above mentioned reviews reported 83% of success in the first 16 cases of sacral neuromodulator implantation in their country; the majority of the cases were indicated for patients with refractory OAB.

An Indian Group from Chandigarh has shown no advantage with the use of isolated whole body 18FDG PET (or combination of PET and CT) versus exclusive computorized tomography for lymph node evaluation in patients with muscle invasive bladder cancer , regarding costs (page 234).

The serum dosage of procalcitonin (PCT) has been popularized for septic patients worldwide. In a South Korean study (page 270), PCT was the strongest predictor of septic chock in patients with urinary sepsis due to acute pyelonephritis, secondary of ureteral calculi (area under curve ROC was 0.929).

Studies reporting minimally invasive procedures in pediatric population are scarce. In page 277, Patel et al., from San Diego, California, reported the use of single site laparoscopic surgery in 44 children (mean age 6.9 years). They used commercial ports, and auxiliary instruments were inserted through fascial punctions. There was only one conversion to open surgery.

Ather and Memmon from Karachi, Pakistan (page 321), reported a so innovative visual pictorial questionnaire to evaluate lower urinary tract symptoms to be applied in illiterate people or among men with low educational level, named VPSS Visual prostate Symptoms Score. VPSS score presented low correlation with maximum urinary flux (Q_max_), suggesting it to be a valid tool for poor and low education populations.

An ancient, but actual discussion is: what is the best approach in open surgery for large glands with benign prostatic hyperplasia, suprapubic or retropubic ? Sakuramoto et al., from ABC School reported better results for the retropubic surgeries, during the residents learning curve.

Returning to children and urinary dysfunction, investigators from Ankara demonstrated that medical therapy is ineffective in more than 50% of kids presenting giggle incontinence (urinary leaks during giggling or laughing while awake). These findings suggest is preferable to avoid drugs in these children.

